# Maternal tobacco, alcohol and caffeine consumption during the perinatal period: A prospective cohort study in Greece during the COVID-19 pandemic

**DOI:** 10.18332/tid/166109

**Published:** 2023-06-16

**Authors:** Maria Tigka, Dimitra Metallinou, Maria Tzeli, Katerina Lykeridou

**Affiliations:** 1 Department of Midwifery, School of Health and Care Sciences, University of West Attica, Athens, Greece; 2 Department of Obstetric Emergency, General and Maternity Hospital 'Helena Venizelou', Athens, Greece

**Keywords:** alcohol, tobacco, caffeine, COVID-19 pandemic, maternal consumption

## Abstract

**INTRODUCTION:**

Low-level knowledge of problematic substance use during the perinatal period may lead to numerous adverse outcomes. We sought to determine maternal tobacco, alcohol and caffeine consumption during the perinatal period during the COVID-19 pandemic.

**METHODS:**

This prospective cohort study recruited women from five Greek maternity hospitals between January and May 2020. Data were collected with a structured questionnaire initially completed by postpartum women during their hospitalization and re-administered via telephone interview in the first, third and sixth month postpartum.

**RESULTS:**

The study sample consisted of 283 women. Smoking rates decreased during pregnancy (12.4%) compared to the pre-pregnancy period (32.9%, p<0.001) and during lactation (5.6%) compared to the antenatal period (p<0.001). The smoking rate increased again after breastfeeding cessation (16.9%) compared to the rate during lactation (p<0.001), but remained lower than the pre-pregnancy rate (p=0.008). Only 1.4% of the women reported breastfeeding cessation due to smoking, whereas those who smoked more during pregnancy were more likely to cease breastfeeding (OR=1.24; 95% CI: 1.05–1.48, p=0.012). Regarding alcohol consumption, it was significantly lower during pregnancy (5.7%), lactation (5.5%) and after breastfeeding cessation (5.2%) compared to the pre-pregnancy period (21.9%, p<0.001 for all correlations). Women who consumed alcohol during lactation were less likely to wean (OR=0.21; 95% CI: 0.05–0.83, p=0.027). Caffeine intake decreased during pregnancy compared to preconception period (p<0.001), while in lactating women it remained at low rates until the 3rd month of follow-up. Caffeine consumption at one month postpartum (β=0.09; SE=0.04, p=0.045) was positively associated with longer duration of breastfeeding.

**CONCLUSIONS:**

Tobacco, alcohol and caffeine consumption decreased in the perinatal period compared to the preconception period. The pandemic may have contributed to the downtrend in smoking and alcohol consumption due to COVID-related restrictions and fear of potential illness. Nevertheless, smoking was associated with reduced duration of breastfeeding and breastfeeding cessation.

## INTRODUCTION

Tobacco, alcohol and caffeine consumption are the most common adopted lifestyle behaviors among women of reproductive age, constituting an ongoing clinical challenge for all healthcare providers. Lack of awareness and low-level knowledge of problematic substance use during the perinatal period may lead to numerous adverse obstetric and neonatal outcomes.

Tobacco use, particularly in the form of cigarette smoking, is a global public health issue that also pertains to pregnant women. According to the report of a Eurobarometer survey in 2020^1^, the prevalence of smoking in Greece (42%) ranks the highest among all European countries, while the rates concerning pregnant women vary substantially among Greek published surveys (13.2–48%)^2-4^. These rates are of great concern, as tobacco consumption during pregnancy has been associated with many complications. Furthermore, tobacco use during the perinatal period is a determinant and influencing factor in the initiation and duration of breastfeeding and is often a cause for maternal-associated breastfeeding cessation^5^.

Another extremely harmful factor during pregnancy and lactation is alcohol consumption. The global incidence of antenatal alcohol use is approximately 10%^6^ and Greece is close to this trend with an estimated incidence of 9.3%^7^ to 11%^8^. Although it is known that alcohol use during pregnancy can cause harm to the developing fetus, the exact amount, pattern and critical period of exposure necessary for harm to occur, are ambiguous. Thus, professional guidance usually focuses on precautionary abstinence and most researchers agree that there is no safe limit at any stage of pregnancy. Alcohol consumption should also be avoided by breastfeeding women as it has been associated with unfavorable consequences on both lactation and the breastfeeding infant. However, research data are insufficient and more studies are needed.

Following on, worldwide, about 80% of the population consumes caffeine products^9^ and as for pregnant women, caffeine is a habitual substance consumed daily by most of them. According to the American Pregnancy Association, the recommended daily dose of caffeine during pregnancy should not exceed 200 mg/day^10^, whereas the WHO recommends no more than 300 mg/day^11^. Regarding the effects of maternal caffeine consumption on the breastfeeding child, a recent study reports that the available cumulative evidence is inadequate and contradictory, thus preventing safe conclusions to be made on this issue^12^.

Several factors determine and influence the onset and duration of maternal substance consumption, including various environmental stressors, such as the recent COVID-19 pandemic. The Greek authorities implemented quarantine and social isolation measures (home confinement, teleworking, work suspension, shop and restaurants closures, reporting to the government via text messages on the reason for leaving home) five days after the WHO declared COVID-19 a global pandemic. We hypothesized that maternal intake of addictive substances would have increased due to restrictions, as a coping strategy, compared to previous studies in Greece. Therefore, the aim of the present study was to assess maternal consumption of tobacco, alcohol and caffeine during the perinatal period during the COVID-19 pandemic. The objectives of the study were to investigate possible associations of the aforementioned lifestyle behaviors with: 1) breastfeeding status at six months postpartum; and 2) breastfeeding duration and cessation.

## METHODS

### Study design

The present study is part of a broader research protocol on addictive substances and medication intake during lactation^13^. It is a prospective cohort study conducted during the COVID-19 pandemic and specifically between January and August 2020. Approval to conduct the study was granted by the Ethics Committees of five tertiary maternity hospitals, three public and two private, in Attica, Greece. The study was conducted according to the guidelines of the Declaration of Helsinki. Women living in Attica and the provinces can obtain maternity services from these hospitals. The research team provided the women with information about the study prior to enrollment to help determine their interest and willingness to serve as research subjects. All participants provided written informed consent.

### Participants and setting

The study recruited mothers who had given birth and were inpatients in the postnatal ward. They had to communicate effectively in the Greek language and have a permanent telephone number for the follow-up procedure. To increase study accuracy, stratified random sampling was employed. Due to COVID-19 limitations, recruitment had to be completed within one month in each maternity hospital. Thus, the final study sample was determined by specific time constraints with an impact on the sampling method of recruitment.

**Figure 1:**
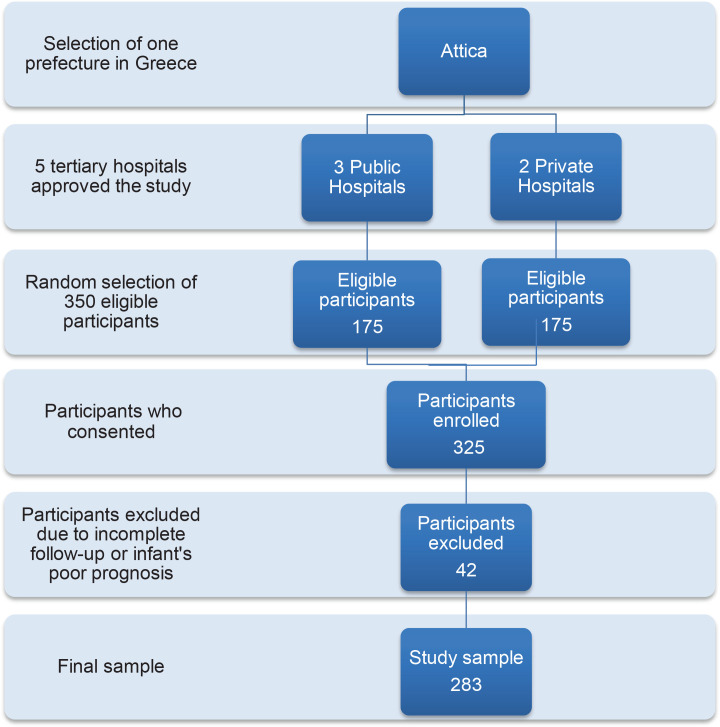
Flowchart of the process of the multistage random sampling method used in the study, Attica, Greece, 2020 (N=283)

Initially, 350 mothers were asked to participate in the study. Of these, 325 agreed and completed the study questionnaire (92.8% response rate). Finally, 283 mothers were enrolled, as 42 were excluded from the study due to: 1) incomplete responses/loss of the questionnaire within the hospitalization (n=17); 2) lack of response at one of the three follow-up time points (n=20); and 3) mothers whose infant was diagnosed with a life-limiting condition or poor prognosis (n=5). The process flowchart of the multistage random sampling method used in the study is illustrated in Figure 1.

### Measures

The survey methodology was used in the form of a 3-part structured questionnaire designed by the first author for the needs of the research. Draft items were created after a thorough literature review^2,9,14^. Prior to the final design, five experts evaluated the content of the questionnaire and checked the clarity of the questions. The draft instrument was pilot-tested during December 2019 on a sample of 50 mothers to identify potential shortcomings and weaknesses of the research tool and protocol (participants in the pilot study were not included in the present study). The final version of the questionnaire was approved by the research team and used open-ended and closed-ended questions.

The first section of the questionnaire included demographic and socioeconomic characteristics (maternal age, ethnicity, marital and employment status, and education level).

The second section of the questionnaire examined maternity and lactation history (type of hospital, parity, maternal body mass index before pregnancy, weight gain during pregnancy, gestational age, mode of delivery, newborn’s gender and birth weight, 6-month breastfeeding follow-up and breastfeeding duration).

The third section of the questionnaire included lifestyle trends (tobacco, alcohol and caffeine consumption before conception, during pregnancy and the postpartum period). Tobacco refers to smoking of cigarettes, either manufactured or roll-your-own, which was measured in pieces per day. E-cigarettes were an option in the questionnaire, but none of the women reported their use. Alcohol was measured in units per week. In Greece, the volume of a glass is used to define a unit of alcohol. Thus, 1 can of beer (330 mL), 1 glass of wine (140 mL) and 1 shot of distilled alcohol (40 mL) were considered as a unit of alcohol. Caffeine consumption was calculated as the average intake of caffeine (mg/day) from food and beverage sources (coffee, tea, carbonated soft drinks, energy drinks, and chocolate).

A follow-up telephone call was made at 1, 3 and 6 months after hospital discharge. The definitions of full breastfeeding (FBF) and mixed breastfeeding (MBF) followed the WHO classification^15^.

### Data collection

Eligible mothers were informed by the first author about the purpose and nature of the study one day after childbirth and were informed that the questionnaire should be completed before discharge from the maternity hospital (the national average stay in the postpartum ward is four days). This provided sufficient time for all potential participants to consider whether they were interested in proceeding with the consent form. The recruitment process was consistent with the recognition of all ethical considerations and there were no direct personal benefits of participating in this study.

Once mothers voluntarily agreed to participate, they were given an envelope containing the study questionnaire and an informed consent form. The questionnaire was distributed in person by the research team in order to avoid systematic handling errors, to provide explanations to participants when necessary, and to achieve clarity of responses.

Upon completion, the relevant documents were enclosed in an envelope and returned to the responsible authors in order to maintain anonymity and confidentiality of the data. At one, three and six months postpartum, mothers were followed-up by telephone interviews to obtain information on tobacco, alcohol, and caffeine consumption. Additionally, breastfeeding status and reasons for discontinuation of breastfeeding were investigated. The follow-up response rate was 93%. Coding of all participants was automatically generated from the database used to maintain de-identification.

### Statistical analysis

Quantitative variables were expressed as mean and standard deviation (SD) or median and interquartile range (IQR). Qualitative variables were expressed as absolute and relative frequencies. The Mann-Whitney test was used to compare continuous variables between two groups. The Wilcoxon signed test and the McNemar test were used to compare participants’ habits between time points. Spearman correlations coefficients were used to investigate the correlation between two continuous variables. Stepwise multiple linear regression analysis was used (p for entry=0.05, p for removal=0.10) with duration of breastfeeding as the dependent variable. Adjusted regression coefficients (β) with standard errors (SE) were calculated from the results of the linear regression analyses. For the linear regression analysis, the dependent variable was transformed into a logarithm. Stepwise logistic regression analysis was used (p for entry=0.05, p for removal=0.10) to find independent factors associated with breastfeeding cessation at 6 months postpartum. Adjusted odds ratios (AOR) with 95% confidence intervals (95% CI) were calculated from the results of the logistic regression analysis. A sample size of 280 participants would provide 99% power to perform a logistic regression analysis with breastfeeding cessation as the dependent variable, at a significance level of 0.05, and determine an estimated odds ratio of at least 2.0. All reported p values are two-tailed. Statistical significance was set at p<0.05 and analyses were performed using SPSS statistical software (version 22.0).

## RESULTS

The study sample consisted of 283 women with a mean age of 33.3 years (SD=5.1). Most participants were married (93.6%) and verified Greek nationality (90.1%). Almost half of the sample (45.6%) were university graduates and 77.4% were professionally active before pregnancy. Maternal sociodemographic characteristics are broadly presented in Table 1.

**Table 1:** Maternal sociodemographic characteristics, Attica, Greece, 2020 (N=283)

*Characteristics*	*n (%)*
**Age** (years), mean ± SD	33.3 ± 5.1
**Nationality**	
Greek	255 (90.1)
Other	28 (9.9)
**Region of residence**	
Attica	218 (77.0)
Provinces	65 (23.0)
**Education level**	
Primary to high school	63 (22.3)
College	57 (20.1)
University	129 (45.6)
Postgraduate studies	34 (12.0)
**Employment before pregnancy**	219 (77.4)
**Family status**	
Unmarried	18 (6.4)
Married	265 (93.6)

Maternal characteristics related to pregnancy, childbirth and breastfeeding are shown in Table 2. Nearly half of the participants were primiparous (50.9%) and delivered in a public hospital (52.3%). Few women gave birth to twins (4.9%). The mean gestational age for all women was 38.3 weeks (SD=1.5). Interestingly, a relatively high percentage of cesarean section (69.6%) and epidural anesthesia during labor (91.8%) was observed. Finally, the women studied had a strong intention to breastfeed (91.8%). Nevertheless, 52.3% had ceased breastfeeding at 6 months postpartum. The median duration of breastfeeding was estimated at 150 days (IQR : 30–181).

**Table 2:** Maternal characteristics related to pregnancy, childbirth and breastfeeding, Attica, Greece, 2020 (N=283)

*Characteristics*	*n (%)*
**Type of maternity hospital**	
Private	135 (47.7)
Public	148 (52.3)
**Parity**	
Multipara	139 (49.1)
Primipara	144 (50.9)
**Pregnancy**	
Single	269 (95.1)
Twin	14 (4.9)
Gestational age (weeks), mean ± SD	38.3 ± 1.5
**Mode of delivery**	
Vaginal delivery	86 (30.4)
Cesarian section	197 (69.6)
Weight gain during pregnancy (kg), mean ± SD	13.0 ± 6.2
BMI (kg/m^2^ ) before pregnancy, mean ± SD	24.3 ± 5.0
**BMI before pregnancy**	
Underweight	15 (5.3)
Normal	169 (59.7)
Overweight	63 (22.3)
Obese	36 (12.7)
**Anesthesia during labor**	
Epidural	259 (91.8)
Local	10 (3.5)
General	13 (4.6)
**Intention to breastfeed**	259 (91.8)
**Neonatal birth weight** (g), mean ± SD	3132.4 ± 473.8
**Sex of the newborn**	
Female	123 (43.5)
Male	160 (56.5)
**Breastfeeding at 6 months postpartum**	
Full/mixed breastfeeding	135 (47.7)
Cessation	148 (52.3)
Days of breastfeeding, median (IQR)	150 (30–181)

Information on participants’ tobacco, alcohol and caffeine consumption habits during the perinatal period is extensively presented in Table 3. Smoking rates decreased significantly during pregnancy (12.4%) compared to the pre-pregnancy period (32.9%, p<0.001) and during lactation (5.6%) compared to the antenatal period (p<0.001). In contrast, the smoking rate increased significantly after breastfeeding cessation (16.9%) in comparison to the rate during lactation (p<0.001), but remained significantly lower than the pre-pregnancy rate (p=0.008). Only 1.4% of the women reported breastfeeding cessation due to smoking.

**Table 3:** Participants’ tobacco, alcohol and caffeine consumption habits, Attica, Greece, 2020 (N=283)

*Tobacco and Alcohol*		*Mean ± SD*	*Median (IQR)*
**Smoking before pregnancy**		93 ± 32.9	
Daily number of cigarettes^a^		12.2 ± 8.1	10 (6–20)
**Smoking during pregnancy**		35 ± 12.4	
Daily number of cigarettes^a^		6.1 ± 4.9	5 (2–10)
**Smoking during lactation**		15 ± 5.6	
Daily number of cigarettes^a^		3.3 ± 2.9	2 (2– 3)
**Smoking after breastfeeding cessation**		41 ± 16.9	
Daily number of cigarettes^a^		10.2 ± 5.9	10 (6–15)
**Alcohol consumption before pregnancy**		62 ± 21.9	
Weekly alcohol units^b^		2 ± 1.5	1 (1– 2)
**Alcohol consumption during pregnancy**		16 ± 5.7	
Weekly alcohol units^b^		1.4 ± 0.6	1 (1– 2)
**Alcohol consumption during lactation**		15 ± 5.5	
Weekly alcohol units^b^		1.3 ± 0.6	1 (1– 1)
**Alcohol consumption after breastfeeding cessation**		13 ± 5.2	
Weekly alcohol units^b^		1.7 ± 0.9	1 (1– 2)
***Caffeine (mg/day)***	***n (%)***	***Mean ± SD***	***Median (IQR)***
Before pregnancy	279 (98.6)	144.7 ± 128.7	101 (58–190)
During pregnancy	245 (86.6)	57.3 ± 61.8	30 (4–94)
On day 4 postpartum	189 (63.8)	38 ± 50.2	6 (0–90)
At 1 month of breastfeeding	218 (73.7)	44.8 ± 55.2	7 (0–90)
At 3 months of breastfeeding	244 (79.8)	51.1 ± 57.8	16 (2–92)
At 6 months of breastfeeding	260 (84.8)	53.9 ± 56.2	28 (3–92)
After breastfeeding cessation	275 (94.7)	105.1 ± 80.3	94 (28–152)

a Refers to those who were smoking.

b Refers to those who consumed alcohol.

Regarding alcohol consumption, it was significantly lower during pregnancy (5.7%), lactation (5.5%) and after breastfeeding cessation (5.2%) compared to the pre-pregnancy period (21.9%, p<0.001 for all correlations). Moreover, alcohol consumption rates did not differ significantly among pregnancy, lactation and after breastfeeding cessation (p>0.05).

Caffeine consumption decreased significantly during pregnancy compared to the pre-pregnancy period (p<0.001). On day 4 postpartum, caffeine consumption was found to be significantly lower in comparison with the antenatal period (p<0.001) and remained at similar levels up to 3 months postpartum (p=0.078). Caffeine consumption increased significantly again at 6 months postpartum (p=0.011) and after breastfeeding cessation (p=0.028).

**Figure 2:**
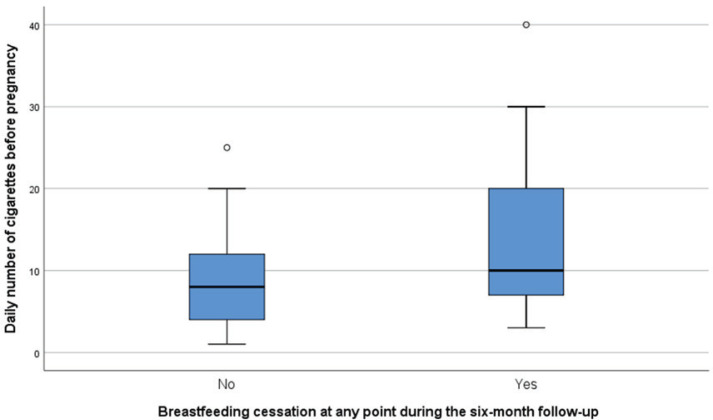
The daily number of cigarettes before pregnancy in association with breastfeeding cessation at any point during the six-month follow-up, Attica, Greece, 2020 (N=283)

Women who discontinued breastfeeding at any point during the six-month follow-up smoked significantly more before (p=0.045) (Figure 2) and during pregnancy (p=0.001), consumed significantly less alcohol during breastfeeding (p=0.017) and less caffeine on day 4 (p=0.019) and 1 month postpartum (p=0.009) compared to women who continued breastfeeding at six months (Table 4). Moreover, longer duration of breastfeeding was significantly associated with: 1) fewer daily number of cigarettes before pregnancy, during pregnancy and after breastfeeding cessation; 2) more units of alcohol during lactation and fewer units after breastfeeding cessation; and 3) greater caffeine consumption on day 4 postpartum and at 1 month of breastfeeding (Supplementary file Table 1).

**Table 4:** Daily number of cigarettes, weekly alcohol units and daily caffeine consumption associated with breastfeeding at 6 months postpartum, Attica, Greece, 2020 (N=283)

*Consumption*	*Breastfeeding at 6 months postpartum*				p*
	*Full/mixed*		*Cessation*		
	*Mean ± SD*	*Median (IQR)*	*Mean ± SD*	*Median (IQR)*
**Daily number of cigarettes**					
Before pregnancy	2.66 ± 5.47	0 (0–2)	5.24 ± 8.59	0 (0–9)	0.045
During pregnancy	0.21 ± 1.14	0 (0–0)	1.26 ± 3.43	0 (0–0)	0.001
During lactation	0.16 ± 0.97	0 (0–0)	0.21 ± 1.05	0 (0–0)	0.439
After breastfeeding cessation	-	-	2.86 ± 5.55	0 (0–3)	-
**Weekly alcohol units**					
Before pregnancy	0.46 ± 1.01	0 (0–1)	0.42 ± 1.13	0 (0–0)	0.262
During pregnancy	0.11 ± 0.42	0 (0–0)	0.05 ± 0.27	0 (0–0)	0.085
During lactation	0.10 ± 0.35	0 (0–0)	0.04 ± 0.28	0 (0–0)	0.017
After breastfeeding cessation	-	-	0.15 ± 0.55	0 (0–0)	-
**Caffeine** (mg/day)					
Before pregnancy	146.09 ± 142.03	98 (43–189)	143.38 ± 115.71	106 (67–194.5)	0.536
During pregnancy	53.60 ± 60.49	25 (4–92)	60.73 ± 62.92	40.5 (4–98)	0.402
On day 4 postpartum	41.27 ± 50.32	7 (0–90)	34.45 ± 50.01	3 (0–90)	0.019
At 1 month of breastfeeding	49.26 ± 56.40	15 (2–92)	39.32 ± 53.5	5.5 (0–90)	0.009
At 3 months of breastfeeding	51.53 ± 56.56	22 (2–92)	50.24 ± 61.2	8.5 (0–91)	0.321
At 6 months of breastfeeding	54.93 ± 56.07	40 (3–92)	44.88 ± 58.65	5 (1.5–91)	0.332
After breastfeeding cessation	-	-	107.35 ± 80.01	95 (40–161)	-

* Mann-Whitney test.

Primiparas (AOR=1.79; 95% CI: 1.07–2.98, p=0.025) and women who smoked more during pregnancy (AOR=1.24; 95% CI: 1.05–1.48, p=0.012) were significantly more likely to cease breastfeeding. In contrast, women who consumed alcohol during lactation were less likely to wean (AOR=0.21; 95% CI: 0.05–0.83, p=0.027). Higher neonatal birth weight was associated with a lower probability of discontinuing breastfeeding (AOR=0.90; 95% CI: 0.85–0.95, p<0.001). Finally, birth weight (β=0.02, SE=0.01, p<0.001), marital status (β=0.34; SE: 0.10, p=0.001) and caffeine consumption at one month postpartum (β=0.09; SE=0.04, p=0.045) were positively associated with longer duration of breastfeeding (Table 5).

**Table 5:** Multiple regression results with stepwise method, Attica, Greece, 2020 (N=283)

*Dependent variable*	*Independent variable*	*AOR (95% CI)*	*p*
**Discontinuation of breastfeeding**	Daily number of cigarettes during pregnancy	1.24 (1.05–1.48)	0.012
	Neonatal birth weight (g)	0.90 (0.85–0.95)	<0.001
	Alcohol consumption during lactation (yes vs no)	0.21 (0.05–0.83)	0.027
	Parity (primipara vs multipara)	1.79 (1.07–2.98)	0.025
		β (SE)*	***p***
**Days of breastfeeding**	Daily number of cigarettes during pregnancy	-0.03 (0.01)	0.002
	Caffeine consumption at 1 month postpartum (mg/day)	0.09 (0.04)	0.045
	Family status (married vs unmarried)	0.34 (0.10)	0.001
	Neonatal birth weight (g)	0.02 (0.01)	<0.001

AOR: adjusted odds ratio. Logistic regression was conducted for breastfeeding cessation and linear regression for the duration of breastfeeding. In the latter case, logarithmic transformations of the dependent variable were used.

* Regression coefficient (Standard Error).

## DISCUSSION

The present study, to our knowledge, is the first Greek study during the pandemic to report on tobacco, alcohol and caffeine consumption during the perinatal period and demonstrate associations with breastfeeding status, duration and cessation. In addition, the present study is the first to document alcohol consumption during lactation and caffeine use during the perinatal period in the Greek population.

Our results contradict the initial hypothesis that maternal intake of the aforementioned substances would have increased due to pandemic restrictions. This hypothesis was based on later studies that investigated the general population and found an increase in smoking and alcohol consumption during lockdown, presumably a strategy used to cope with emotions like anxiety and stress^16,17^. Previous studies have examined in the general population the association between tobacco and alcohol consumption with the severity of COVID-19 disease^18,19^ and few researchers have addressed the issue of maternal tobacco and alcohol use during the COVID-19 pandemic^20-23^.

Regarding maternal tobacco consumption, the results of the present study reveal that smoking rates decreased significantly during the perinatal period compared to pre-pregnancy, and during lactation compared to the antenatal period. However, rates increased again after breastfeeding cessation but remained significantly lower in comparison with pre-pregnancy rates. This finding is consistent with a previous study^24^ and suggests that women may recognize the adverse effects of smoking and be more motivated during the perinatal period to reduce tobacco consumption. Furthermore, longer duration of breastfeeding was associated with lower number of cigarettes per day in the pre-pregnancy, antenatal and postnatal period. Smoking before pregnancy was a risk factor for early breastfeeding cessation, which is in agreement with other studies^25-27^.

Comparing our findings with earlier Greek studies conducted before the pandemic, we observe that smoking rates during the perinatal period have decreased. Specifically, the prevalence of prepregnancy tobacco use in our study was estimated at 32.9% in contrast to 36.1%^2^ and 41.4%^3^, rates evolving from recent Greek studies. Tobacco use during pregnancy was calculated at 12.4%, which is lower than the 13.2%^2^, 19.7%^3^ and 26.3%^4^ reported by other Greek research teams. Tobacco consumption decreased even more during lactation in our study, reaching the rate of 5.6%, which is much lower than the 22% reported by Iliodromiti et al.^28^ in the National Breastfeeding Study in Greece. Previous studies conducted during the pandemic concluded that pregnant and lactating women were less likely to report tobacco use compared to counterparts one year before^20,23^, and that smoking cessation rates in pregnant women were unaffected by the COVID-19 pandemic^21^. Undoubtedly, the appearance of the coronavirus and fear of potential illness during the vulnerable perinatal period may have contributed to the reduction of addictive substance abuse.

Alcohol was the second addictive substance studied in this research. Our findings revealed that alcohol consumption decreased significantly during the perinatal period compared to the preconception period but the rates during pregnancy, breastfeeding and after breastfeeding cessation did not differ much among them (5.2–5.7%). Only minor changes were observed in alcohol consumption patterns, which may be due to light drinking, a case that is also stated by Tran et al.^29^. However, this finding contradicts a Korean study which reported that alcohol consumption rates were significantly higher during pregnancy compared to breastfeeding, possibly due to the fact that women were unaware of their status during early pregnancy^30^.

Another interesting finding in the present study was that longer duration of breastfeeding was associated with more units of alcohol consumption. This is consistent with other studies^31,32^, while other researchers found no correlation^14,27^. We hypothesize that the increased consumption of alcohol (mainly beer in the present study) resulted from the mistaken belief that alcohol stimulates milk production^6^. Another possible explanation may be that the lift of confinement restrictions because of the pandemic coincided with the period when the first participants were in the 3rd to 6th month postpartum, and therefore the increased social interaction may have led to alcohol consumption. Comparing our results with previous Greek studies carried out before the COVID-19 era, we observed lower rates of alcohol consumption during pregnancy. In particular, the alcohol rate in the present study reached 5.7%, which is considerably lower in comparison with the 9.3% and 11% mentioned by other Greek studies^7,8^. The decrease in alcohol consumption among pregnant women reported also by other research teams^22,23^, could be attributed to COVID-related restrictions^22^.

As far as caffeine use is concerned, our results showed that a high proportion of women (98.6%) consumed caffeine products before conception, which decreased during pregnancy (86.6%) and even more on day 4 postpartum (63.8%), followed by an increase in the first (73.7%), third (79.8%) and sixth (84.8%) month after delivery and after breastfeeding cessation (94.7%). In the US, 80% of pregnant women continue to consume coffee during pregnancy, a prevalence very close to that of our study^33^, while European countries report lower values during pregnancy (France: 47.1%, Italy: 42.3%, Finland: 31%)^34^. Current research on caffeine consumption during breastfeeding focuses mainly on adverse effects on infants^35,36^, rather than on prevalence and maternal outcomes in relation to the extent of breastfeeding exclusivity and duration. Especially in the Greek population, there is no previous study recording coffee consumption neither during pregnancy nor during breastfeeding, so no conclusions can be drawn about the effect of the COVID-19 era on maternal caffeine consumption.

Finally, we believe that the study’s importance lies also in the finding that the maternal characteristics of the study sample, compared with data from the Greek Statistical Authority (GSA) and the Hellenic Authority for Higher Education (HAHE), were a significant reflection of the Greek female population^37,38^. In this study more specifically, 45.6% of the women were university graduates, 93.6% were married, 90.1% had Greek ethnicity and the mean age was 33.27 ± 5.11 years. Based on data from the HAHE, 50% of women aged 25–34 years had tertiary education degrees in 2020, while GSA data showed that 90.8% of the women who gave birth during 2020 were married, 85.2 % were Greek and the mean age was 31.6 years.

### Strengths and limitations

The present study contributes to the better understanding of maternal consumption of addictive substances in the Greek population during the COVID-19 pandemic. The importance of our work lies in the prospective longitudinal design and the high response rates achieved both during hospitalization and the follow-up procedure. The fact that the questionnaire was distributed in person by the researchers helped to gain trust in research participants and provide occasional explanations and clarifications, increasing the reliability and thus the strengths of our study. Furthermore, the fact that the data were obtained from five tertiary referral maternity hospitals and maternal characteristics are consistent with national statistics, renders the sample more representative. However, the generalization of the findings to a national level is limited since the sample included mothers from only one major region, although they came to these hospitals from smaller regions as well. Another limitation is that the final study sample was determined by specific time constraints due to COVID-19 restrictions, which undoubtedly had an impact on the small sample size. Finally, data were collected through self-reports which might have led to underestimation or overestimation of the prevalence and amount of substance use.

### Recommendations for clinical practice and future research

Pregnant women are more amenable to adopting new and healthier habits than any other group in the community. Pregnancy and lactation can act as incentives for the cessation of addictive substances. Healthcare providers should take advantage of these periods to disseminate public health messages aimed at improving perinatal outcomes. During antenatal care and parental education, healthcare professionals should provide mothers with information on the adverse effects of addictive substance use during the perinatal period, taking into account scientific data from evidence-based information sources and referring to guidelines from scientific committees. Additionally, women should be informed about the specialized professional support available in relation to smoking cessation, the evidence-based interventions for smoking cessation in the context of routine antenatal care, and the accessible counseling and psychosocial units.

All maternity units should be staffed by specially trained professionals who will identify high-risk patients and provide appropriate intervention. In addition, all maternity units should have specific care and intervention departments for the cessation of addictive substances during the perinatal period, as well as follow-up departments to maintain healthy lifestyle habits and prevent relapse.

More research should be carried out to improve early intervention programs in order to increase their effectiveness. The importance of primary prevention of smoking and alcohol consumption, which can be achieved through health education programs in schools and family planning clinics, should be emphasized. The introduction of universal screening protocols for women in reproductive age is also of paramount importance. Finally, further research is needed to examine the impact of substance use on the initiation, duration and breastfeeding cessation.

## CONCLUSIONS

Tobacco, alcohol and caffeine consumption decreased in the perinatal period compared to the preconception period. The downtrend in smoking and alcohol consumption compared to earlier Greek studies conducted before the COVID-19 era suggests that this unprecedented time of human history may have contributed to the reduction of substance consumption probably due to the fear of contamination during the sensitive perinatal period. Nevertheless, smoking was associated with reduced duration of breastfeeding and breastfeeding cessation indicating the need for implementing effective strategies to intensify maternal smoking cessation during pregnancy and postpartum abstinence. Inclusion of the partner and family in smoking cessation programs, in our opinion, would sustain the positive impact of these programs and therefore fully contribute to public health.

## Data Availability

The data supporting this research are available from the authors on reasonable request.
