# Supplementary material for: Maternal tobacco, alcohol and caffeine consumption during the perinatal period: A prospective cohort study in Greece during the COVID-19 pandemic
Source: Tob Induc Dis. 2023 Jun 16;21:80. doi: 10.18332/tid/166109 (PMC10273827; doi:10.18332/tid/166109)
Supplement: Supplementary file 1 [file TID-21-80-s1.pdf]

Table S1

Spearman correlation coefficients of days of breastfeeding with daily number of cigarettes, weekly alcohol units and daily caffeine consumption (Attica/Greece, 2020; N=283)

	Days of breastfeeding	
	rho (ρ)	P
**Daily number of cigarettes**		
before pregnancy	-0.13	0.028
during pregnancy	-0.24	<0.001
during lactation	-0.05	0.396
after breastfeeding cessation	-0.40	<0.001
**Weekly alcohol units**		
before pregnancy	0.04	0.476
during pregnancy	0.07	0.235
during lactation	0.13	0.036
after breastfeeding cessation	-0.20	0.001
**Caffeine (mg/day)**		
before pregnancy	0.02	0.778
during pregnancy	-0.07	0.275
on day 4 postpartum	0.15	0.018
at 1 month of breastfeeding	0.18	0.004
at 3 months of breastfeeding	0.06	0.424
at 6 months of breastfeeding	-0.01	0.942
after breastfeeding cessation	0.02	0.827

© **2023 Tigka M. et al**.
